# *Elsholtzia yajiangensis* (Lamiaceae), a New Species From Sichuan, China

**DOI:** 10.1002/ece3.73817

**Published:** 2026-06-15

**Authors:** Li‐Jia Liu, Yan Wang, Hai‐Lei Zheng, Yao Luo, Hong Jiang, Qiang Wang

**Affiliations:** ^1^ State Key Laboratory of Plant Diversity and Specialty Crops, Institute of Botany Chinese Academy of Sciences Beijing China; ^2^ National Botanical Garden, Institute of Botany Chinese Academy of Sciences Beijing China; ^3^ College of Life Sciences University of Chinese Academy of Sciences Beijing China; ^4^ Chengdu Institute of Biology Chinese Academy of Sciences Chengdu China

**Keywords:** *Elsholtzia*, Lamiaceae, morphometrics, new species, taxonomy

## Abstract

*Elsholtzia* Willd. is the largest genus in tribe Elsholtzieae (Lamiaceae) and has notable medicinal and economic value in East Asia. During a field survey in Sichuan Province, China, we discovered a morphologically distinct population of *Elsholtzia* resembling *E. lamprophylla* but differing in leaf size, indumentum, inflorescence type, and bract shape. To determine its taxonomic status, we conducted phylogenomic analyses using 1115 single‐copy nuclear genes and 240 plastid loci from 45 accessions, representing major lineages of *Elsholtzia*. Both nuclear coalescent and concatenation‐based trees consistently placed the new species in Clade I (*Ser. Fruticosae*) with strong support, while plastid data revealed a close relationship with several *Ser. Fruticosae* members, suggesting possible hybridization or incomplete lineage sorting. Morphometric analyses of 12 vegetative and floral traits clearly separated the new species from *E. lamprophylla*. Based on morphological and phylogenomic evidence, we describe it as *Elsholtzia yajiangensis* Y. Wang, L. J. Liu & Q. Wang, sp. nov. It is characterized by its secund spikes, broadly lanceolate bracts, strigose leaves with 5–7 lateral veins, and remotely obtuse‐serrate margins. The species is currently known only from the type locality in Yajiang County, Sichuan, and is provisionally assessed as Data Deficient (DD) based on its narrow distribution and ongoing threats. This discovery highlights that the species diversity of *Elsholtzia* in the Hengduan Mountains remains underestimated.

## Graphical abstract

Adds to the understanding of species diversity in the taxonomically challenging genus Elsholtzia, particularly in the biodiversity hotspot of the Hengduan Mountains. Demonstrates the utility of combining shallow whole‐genome sequencing and morphometrics in resolving species boundaries in closely related shrubby taxa. Highlights that the diversity of Elsholtzia in China may still be underestimated, encouraging further exploration.

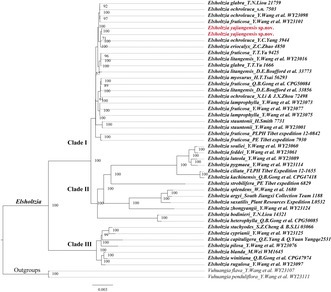

## 1 Introduction

*Elsholtzia* Willd. (Nepetoideae, Lamiaceae), the largest genus within the tribe Elsholtzieae, comprises approximately 40 species widely distributed across East Asia (Li et al. 2017; Wang et al. 2024; Wu and Li 1977). Species of *Elsholtzia* are utilized in various fields, including medicine, spices, and food ingredients, underscoring their significant economic importance (Guo et al. 2012). It is an important source of folk traditional Chinese medicine, mainly used to relieve the symptoms of cold, fever, pneumonia, and so on (Chen et al. 2022). In recent years, the discovery of three new species in China suggests that the diversity of the genus may still be underestimated (Jin et al. 2022; Pu et al. 2012; Xiang and Liu 2012).

During a field survey in Yajiang County, Sichuan Province, China, in 2024, we encountered a putative new species of *Elsholtzia*. It resembles *Elsholtzia lamprophylla* C. L. Xiang & E. D. Liu but exhibits notable differences in leaf size, indumentum, inflorescence morphology, and bract shape (Xiang and Liu 2012). Following meticulous examination of herbarium specimens, literature review, and detailed morphological and phylogenetic analyses, we confirm this collection as a distinct new species, which we describe here as *Elsholtzia yajiangensis*, named after its type locality.

## 2 Methods and Materials

### 2.1 Taxon Sampling and Sequencing

**FIGURE 1:**
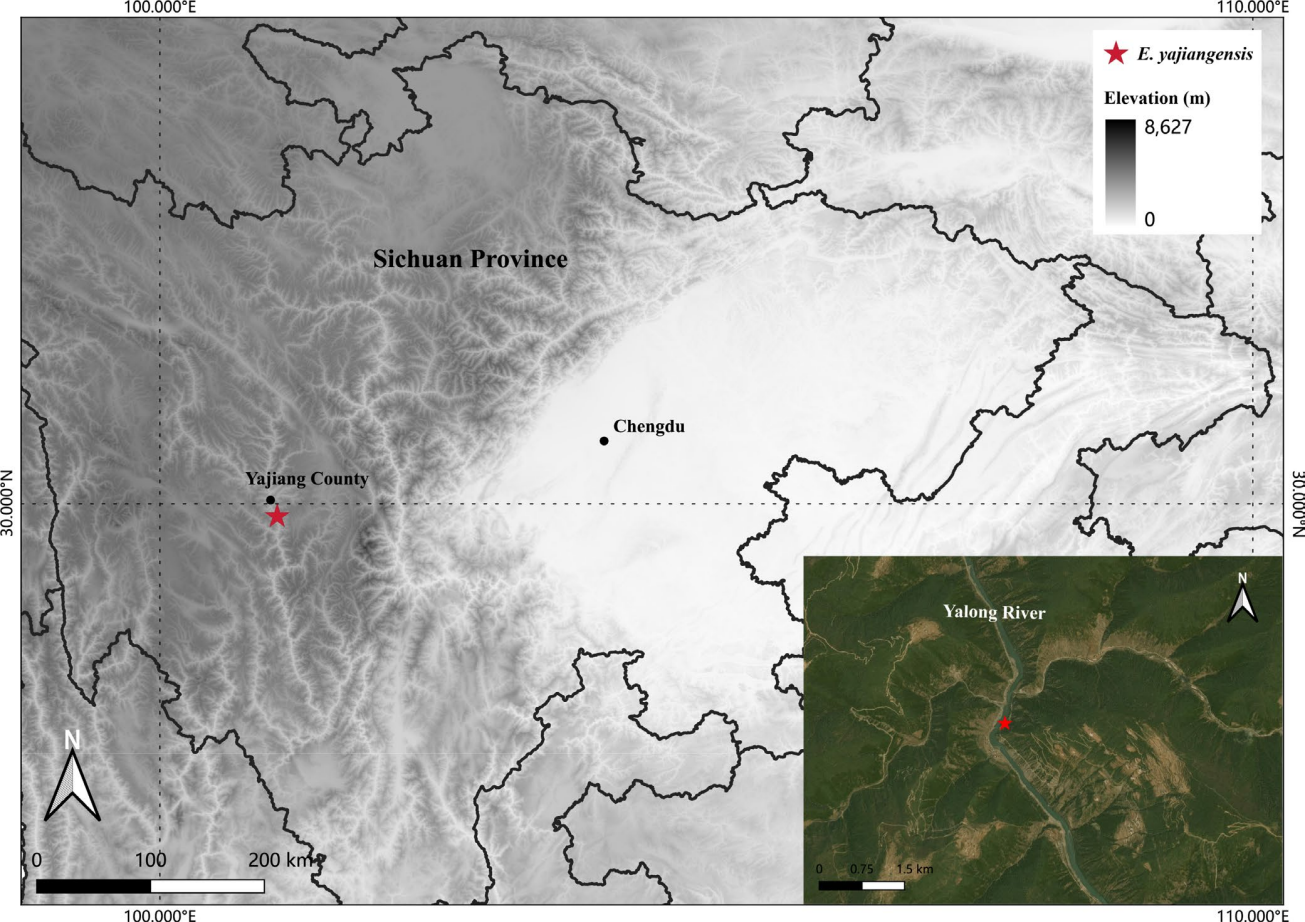
Distribution of *Elsholtzia yajiangensis* sp. nov. (red stars) in Sichuan, China. A detailed view of the terrain and rivers is shown in the bottom right‐hand corner.

Materials of *E. yajiangensis* were collected during fieldwork in 2024. This population is located in southern Yajiang County, along the riverbanks of the middle reaches of the Yalong River in the Hengduan Mountains, at elevations ranging from approximately 2500–2700 m (Figure 1). Following Wang et al. (2024), *Vuhuangia* was selected as the outgroup. A total of 45 samples were included in phylogenetic analysis, comprising 29 species, 43 samples of *Elsholtzia* (including two *E. yajiangensis*) and two species of *Vuhuangia*. Among the materials subjected to whole‐genome sequencing (WGS), 25 samples were silica gel‐dried leaves collected in the field, and 20 samples were from herbarium specimens deposited in the herbarium of the Institute of Botany, Chinese Academy of Sciences (PE) with appropriate permissions. Collection details for all taxa are provided in Table 1. In addition, transcriptomic data for 10 accessions were analyzed, including representatives from all major Elsholtzieae clades (8 samples) as well as one species each from Mentheae and Ocimeae, all downloaded from NCBI.

**TABLE 1:** Voucher information and accession no. of samples used in this study.

Taxon	Voucher	Location	Sample form for WGS	WGS	Plastome
*Elsholtzia argyi*	South Jiangxi Collection Team 1188 (PE)	Chongyi, Jiangxi, China	Specimen leaves	SRR28959273	PP357185
*Elsholtzia blanda*	M.Wei WM1645 (PE)	Mêdog, Xizang, China	Silica gel‐dried leaves	SRR28959272	PP357210
*Elsholtzia bodinieri*	T.N.Liou 14321 (PE)	Kunming, Yunnan, China	Specimen leaves	SRR28959250	PP357159
*Elsholtzia capituligera*	Q.E.Yang & Q.Yuan Yangqe2531 (PE)	Dêqên, Yunnan, China	Specimen leaves	SRR28959228	PP357162
*Elsholtzia ciliata*	FLPH Tibet Expedition 12‐1655 (PE)	Bomê, Xizang, China	Specimen leaves	SRR28959195	PP357171
*Elsholtzia cyprianii*	Y.Wang et al. WY23125 (PE)	Kangding, Sichuan, China	Silica gel‐dried leaves	SRR28959266	PP357245
*Elsholtzia eriocalyx*	Z.C.Zhao 4850 (PE)	Sichuan, China	Specimen leaves	**SRR38564186**	**PZ392119**
*Elsholtzia feddei*	Y.Wang et al. WY23061 (PE)	Kangding, Sichuan, China	Silica gel‐dried leaves	SRR28959259	PP357233
*Elsholtzia fruticosa1*	PLPH Tibet Expedition 12–0842 (PE)	Cona, Xizang, China	Specimen leaves	SRR28959258	PP357186
*Elsholtzia fruticosa2*	PE Tibet Expedition 7930 (PE)	Gyirong, Xizang, China	Silica gel‐dried leaves	SRR28959257	PP357227
*Elsholtzia fruticosa3*	T.T.Yu 9425 (PE)	Dêqên, Yunnan, China	Specimen leaves	SRR28959256	PP357204
*Elsholtzia fruticosa4*	Q.B.Gong et al. CPG50084 (PE)	Yingjiang, Yunnan, China	Silica gel‐dried leaves	SRR28959255	PP357221
*Elsholtzia fruticosa5*	Y.Wang et al. WY23077 (PE)	Xiangcheng, Sichuan, China	Silica gel‐dried leaves	SRR28959254	PP357236
*Elsholtzia fruticosa6*	Y.Wang et al. WY23101 (PE)	Binchuan, Yunnan, China	Silica gel‐dried leaves	SRR28959208	PP357240
*Elsholtzia glabra1*	T.N.Liou 21759 (PE)	Binchuan, Yunnan, China	Specimen leaves	SRR28959251	PP357161
*Elsholtzia glabra2*	T.T.Yu 1666 (PE)	Yanbian, Sichuan, China	Specimen leaves	SRR28959252	PP357200
*Elsholtzia heterophylla*	Q.B.Gong et al. CPG50085 (PE)	Yingjiang, Yunnan, China	Silica gel‐dried leaves	SRR28959248	PP357214
*Elsholtzia kachinensis*	Q.B.Gong et al. CPG47418 (PE)	Longchuan, Yunnan, China	Silica gel‐dried leaves	SRR28959246	PP357215
*Elsholtzia lamprophylla*	Y.Wang et al. WY23075 (PE)	Xiangcheng, Sichuan, China	Silica gel‐dried leaves	SRR28959245	PP357234
*Elsholtzia lamprophylla*	Y.Wang et al. WY23073 (PE)	Xiangcheng, Sichuan, China	Silica gel‐dried leaves	**SRR38564185**	**PZ392120**
*Elsholtzia litangensis1*	D.E.Boufford et al. 33856 (PE)	Jomda, Xizang, China	Specimen leaves	SRR28959244	PP357202
*Elsholtzia litangensis2*	D.E.Boufford et al. 33773 (PE)	Sêrxü, Sichuan, China	Specimen leaves	SRR28959243	PP357203
*Elsholtzia litangensis3*	Y.Wang et al. WY23016 (PE)	Baiyü, Sichuan, China	Silica gel‐dried leaves	SRR28959242	PP357228
*Elsholtzia luteola*	Y.Wang et al. WY23089 (PE)	Yunlong, Sichuan, China	Silica gel‐dried leaves	SRR28959240	PP357237
*Elsholtzia myosurus*	H.T.Tsai 56293 (PE)	Lanping, Yunnan, China	Specimen leaves	SRR28959238	PP357155
*Elsholtzia ochroleuca1*	X.Li & J.X.Zhou 72498 (PE)	Barkam, Sichuan, China	Specimen leaves	SRR28959237	PP357187
*Elsholtzia ochroleuca2*	s.coll. 7503 (PE)	China	Specimen leaves	SRR28959236	PP357192
*Elsholtzia ochroleuca3*	Y.C.Yang 3944 (PE)	Sichuan, China	Specimen leaves	SRR28959235	PP357193
*Elsholtzia ochroleuca4*	Y.Wang et al. WY23098 (PE)	Binchuan, Yunnan, China	Silica gel‐dried leaves	SRR28959234	PP357239
*Elsholtzia pilosa*	Y.Wang et al. WY23076 (PE)	Xiangcheng, Sichuan, China	Silica gel‐dried leaves	SRR28959232	PP357235
*Elsholtzia pygmaea*	Y.Wang et al. WY23114 (PE)	Kunming, Yunnan, China	Silica gel‐dried leaves	SRR28959231	PP357243
*Elsholtzia rugulosa*	Y.Wang et al. WY23097 (PE)	Yulong, Yunnan, China	Silica gel‐dried leaves	SRR28959227	PP357238
*Elsholtzia saxatilis*	Plant Resources Expedition L0532 (PE)	Song, Henan, China	Specimen leaves	SRR28959226	PP357166
*Elsholtzia souliei*	Y.Wang et al. WY23060 (PE)	Kangding, Yunnan, China	Silica gel‐dried leaves	SRR28959220	PP357232
*Elsholtzia splendens*	W.Wang et al. 1680 (PE)	Fengcheng, Liaoning, China	Specimen leaves	SRR28959219	PP357158
*Elsholtzia stachyodes*	S.Z.Cheng & B.S.Li 03066 (PE)	Mêdog, Xizang, China	Specimen leaves	SRR28959218	PP357157
*Elsholtzia stauntonii1*	Y.Wang et al. WY23001 (PE)	Beijing, China	Silica gel‐dried leaves	SRR28959214	PP357209
*Elsholtzia stauntonii2*	H.Smith 7731 (PE)	Shanxi, China	Specimen leaves	SRR28959215	PP357164
*Elsholtzia strobilifera*	PE Tibet Expedition 5795 (PE)	Lhünzê, Xizang, China	Silica gel‐dried leaves	SRR28959213	PP357224
*Elsholtzia winitiana*	Q.B.Gong et al. CPG47974 (PE)	Mang, Yunnan, China	Silica gel‐dried leaves	SRR28959209	PP357220
*Elsholtzia yajiangensis*	Y.Wang & H.L.Zheng WY24039 (PE)	Yajiang, Sichuan, China	Silica gel‐dried leaves	**SRR38564184**	**PZ392118**
*Elsholtzia yajiangensis*	Y.Wang & H.L.Zheng WY24039 (PE)	Yajiang, Sichuan, China	Silica gel‐dried leaves	**SRR38564183**	**PZ392117**
*Elsholtzia zhongyangii*	Y.Wang et al. WY23124 (PE)	Baiyü, Sichuan, China	Specimen leaves	SRR28959207	PP357244
*Vuhuangia flava*	Y.Wang et al. WY23107 (PE)	Longyang, Yunnan, China	Silica gel‐dried leaves	SRR28959188	PP357241
*Vuhuangia penduliflora*	Y.Wang et al. WY23111 (PE)	Kunming, Yunnan, China	Silica gel‐dried leaves	SRR28959186	PP357242

*Note:* The text in bold indicates data newly uploaded for this study.

Library preparation and shallow whole‐genome sequencing were carried out following Wang et al. (2024). Genomic DNA was extracted using a modified CTAB protocol (Doyle et al. 1987). Paired‐end libraries (150‐bp insert size) were constructed and sequenced on the DNBSEQ‐T7 platform. Adapter trimming and quality control of raw reads were performed with Fastp v0.23.1 (Chen et al. 2018).

### 2.2 Ortholog Identification and Assembly

RNA‐seq reads from 10 samples were assembled into contigs using Trinity v2.15.1 (Grabherr et al. 2011; Haas et al. 2013) under default parameters. The longest isoform per gene was retained to generate a set of unigenes, from which protein‐coding regions were predicted using TransDecoder v5.7.1 (Haas et al. 2013). Putative orthologous genes were identified through Orthofinder v2.5.4 (Emms and Kelly 2019). These orthologues were used as target sequences for gene recovery from WGS data. Prior to assembly, target sequences containing low‐complexity regions were filtered to minimize downstream artifacts such as excessive off‐target mapping, prolonged assembly times, and inflated log file sizes, following recommendations from the HybPiper pipeline. Gene recovery from WGS data was conducted using HybPiper v2.3.1 (Johnson et al. 2016) with the DIAMOND map option. After assembly, the *paralog_retriever* function in HybPiper was used to identify genes with potential paralogs. Genes flagged as containing putative paralogs in more than four samples (i.e., > 5%) were excluded from downstream analyses. For genes with paralog warnings in one to four samples, we further examined the putative paralogs by aligning the sequences and reconstructing gene trees. If putative paralogs from the same sample clustered together, the gene was considered not indicative of an ancient gene duplication and was retained using the primary sequences (denoted as “.main”); otherwise, the gene was discarded. The “.main” sequence represent the contig selected by HybPiper based on either higher read depth or greater sequence similarity to the reference, and is considered appropriate for use in phylogenetic analyses when ancient gene duplication is not evident.

### 2.3 Plastome Assembly and Annotation

*De novo* plastome assembly was performed using the GetOrganelle v1.7.7.0 (Jin et al. 2020). Plastome annotations were carried out with the Plastid Genome Annotator (Qu et al. 2019), using the published plastome of *Elsholtzia ciliata* (Thunb.) Hyland. (GenBank accession no.: PP357171) as reference. Annotated plastomes were then verified and visualized in Geneious (Kearse et al. 2012). The newly acquired plastome sequences for the two *E. yajiangensis*, along with two additional sequences of *Elsholtzia eriocalyx* C.Y.Wu & S.C.Huang and *Elsholtzia lamprophylla* C.L.Xiang & E.D.Liu, have been officially submitted to GenBank (accession numbers in Table 1). The circular plastome maps of *E. yajiangensis* were visualized by the online tool Chloroplot (Zheng et al. 2020). Coding and noncoding regions were extracted using python script “get_annotated_regions_from_gb.py” (https://github.com/Kinggerm/PersonalUtilities/tree/master).

### 2.4 Sequence Alignment and Phylogenetic Analysis

All individual gene matrices were aligned using MAFFT v7.520 (Katoh and Standley 2013) and trimmed using Gblocks v0.91b (Castresana 2000), allowing for up to 50% gaps.

For the nuclear dataset, phylogenetic relationships were reconstructed under both multispecies summary coalescent (MSC) and concatenated approaches. For MSC method, gene trees were estimated using RAxML v8.2.12 (Stamatakis 2014) under the GTRCAT model with 200 bootstrap replicates. ASTRAL v5.7.8 (Mirarab et al. 2014; Zhang et al. 2018) was employed to infer a species tree. For concatenated tree, Maximum likelihood (ML) inference was conducted using IQ‐TREE v2.2.2.6 (Minh et al. 2020) with 1000 ultrafast bootstrap replicates under the partitioning scheme (Chernomor et al. 2016; Hoang et al. 2018). ModelFinder (Kalyaanamoorthy et al. 2017) was employed to define data partitions and select the optimal nucleotide substitution model for each.

The plastid species tree was inferred based on the concatenation method. The trimmed alignments were merged into a concatenated supermatrix, and phylogenetic reconstruction was performed using the same maximum likelihood approach as for the nuclear concatenated dataset.

Topological congruence between trees generated by different methods or datasets was assessed using the normalized Robinson‐Foulds (RF) distance implemented in the ape v5.8‐1 (Paradis and Schliep 2019) and phangorn v2.12.1 (Schliep 2011) packages in R v4.5.1. The RF distance quantifies topological differences by comparing bipartitions between trees, with values ranging from 0 (identical topologies) to 1 (completely different topologies). Prior to comparison, both trees were rooted using *Vuhuangia* as outgroups to ensure topological comparability. Tanglegrams were generated using the phytools package v2.5‐2 (Revell 2024) to visually compare corresponding taxa positions between trees.

### 2.5 Morphological Comparisons

To compare the morphological characteristics between *E. yajiangensis* and its closely related species *E. lamprophylla*, we summarized their key diagnostic traits and selected seven leaf morphological traits and five floral/inflorescence traits for measurement using MATO (Liu et al. 2023). All sampled leaves were collected from mature, flowering individuals to minimize variation associated with developmental stage. These traits include: leaf length (including petiole), leaf width, leaf ratio (leaf length/width), petiole length, petiole ratio (petiole length/leaf length), number of lateral veins, number of serrations (per side), inflorescence (spike) length, bract length, calyx length, corolla length, and spike type (secund = 1, cylindric = 0). Given that both species are narrow endemics with limited population sizes and few available individuals, morphometric measurements were primarily based on type specimens (collection details: *E.D. Liu, C.L. Xiang & X. Nong 2697* and *Y. Wang & H. L. Zheng WY24039*) together with field observation. For each species, 3–5 mature individuals were examined. For each individual, 5–10 organs of the same type (e.g., leaves or flowers) were randomly selected and measured for each morphological trait. After data aggregation, 16 morphometric records were obtained for *E. lamprophylla* and 26 for *E. yajiangensis* for PCA analyses. We acknowledge that the limited sampling inherent to these narrowly distributed species may not fully capture within‐population variation, and therefore the PCA separation should be interpreted together with qualitative morphological differences and other lines of evidence rather than as a standalone basis for species delimitation. Phenological information was inferred from field observations conducted during specimen collection, together with observations from examined specimens.

To better visualize morphological distinctions, the data were divided into three sets for analysis: (1) all 12 traits combined, (2) 7 leaf traits only, and (3) 5 floral traits only. Principal component analysis (PCA) was performed on these morphological datasets using the factoextra v1.0.7 (Kassambara et al. 2020) and tidyverse v2.0.0 (Wickham et al. 2019) packages in R v4.5.1, and the results were plotted accordingly.

## 3 Results and Discussion

### 3.1 Ortholog Identification and Assembly

The four newly sequenced WGS samples (2 *E. yajiangensis*, 1 *E. lamprophylla* and 1 *E. eriocalyx*) generated 12.64–23.42 Gb of raw sequencing data per sample. The two samples of *E. yajiangensis* contain 23.42 Gb and 22.02 Gb of raw data respectively. The remaining 41 samples used in this study yield between 24.29 Gb and 58.89 Gb of raw data. All 45 samples were retained for analysis. Sequencing depth was estimated based on the genome size of the diploid species *Elsholtzia splendens* (Moon et al. 2025).

A total of 1536 single‐copy orthologous genes (SCO) were identified from the 10 assembled transcriptomes. Among the resulting 15,360 sequences (1536 × 10), 4678 were detected as low‐complexity sequences and subsequently removed. As a result, several genes had all 10 sequences filtered out, leaving 1454 genes (comprising 10,682 sequences) as the target set for assembly. Among these genes, between 1141 and 1454 were successfully assembled per sample. After filtering genes with paralog warnings, a total of 1115 genes were retained for downstream phylogenetic analyses.

### 3.2 Plastome Assembly and Annotation

The complete plastome of *E. yajiangensis* was assembled and found to be 151,523 bp in length (Figure S1). Its annotation revealed a total of 130 genes, comprising 8 rRNA genes and 37 tRNA genes. According to Wang et al. (2024), plastome lengths in the tribe Elsholtzieae range from 148,245 bp to 152,704 bp. The plastome size of *E. yajiangensis* is consistent with this reported range. A total of 240 loci were extracted from the 45 plastomes, including 79 exons, 20 introns, 34 RNAs, and 107 intergenic regions. The concatenated matrix contained 123,430 sites.

### 3.3 Phylogenetic Analyses

**FIGURE 2:**
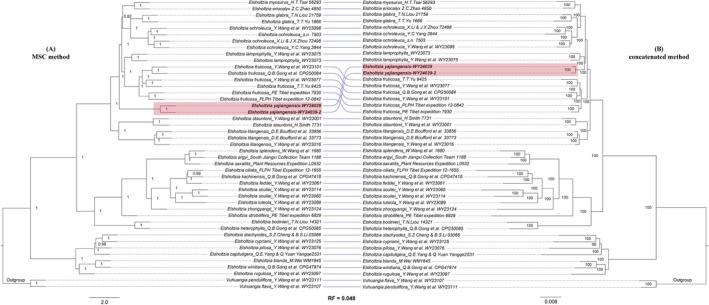
Phylogenetic relationships of *Elsholtzia* inferred from nuclear gene data. (A) Species tree reconstructed from 1115 single‐copy nuclear genes under the multispecies coalescent model using ASTRAL. Node support values represent local posterior probabilities (LPP). (B) Maximum likelihood tree inferred from the concatenated nuclear matrix with IQ‐TREE under a partitioned substitution model. Ultrafast bootstrap support (UFBoot) from 1000 replicates is shown at nodes. Lines connect corresponding taxa between the two trees; the Robinson–Foulds (RF) distance between the topologies is 0.048, indicating a high degree of congruence.

**FIGURE 3:**
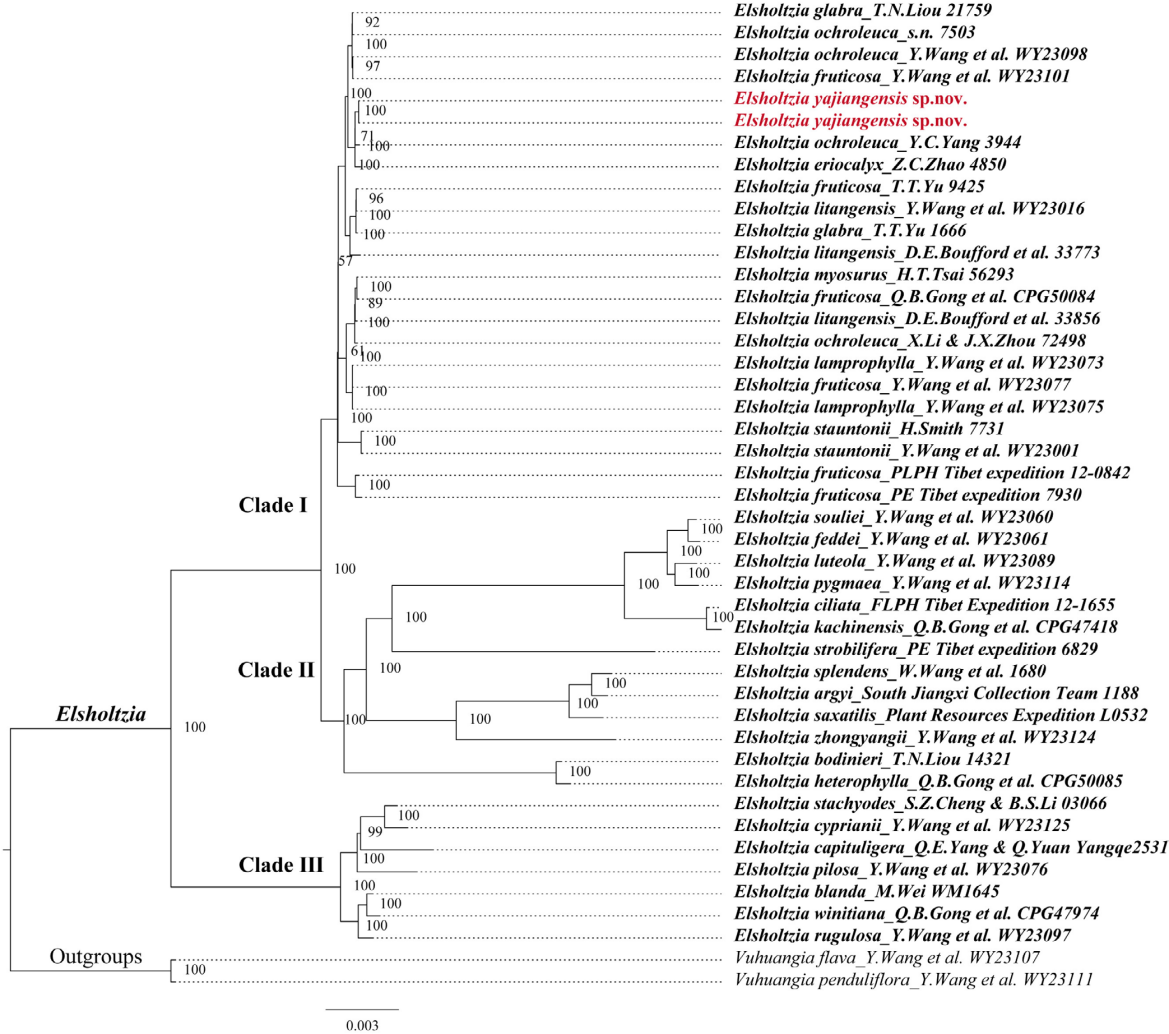
Maximum likelihood phylogeny of *Elsholtzia* based on the plastid genome. The tree was inferred from a concatenated matrix of 240 plastid loci (including exons, introns, and intergenic spacers; 123,430 aligned sites) using IQ‐TREE with 1000 ultrafast bootstrap replicates. Bootstrap support values are shown at nodes.

All three phylogenetic reconstructions consistently supported the division of *Elsholtzia* into three stable clades, with *E. yajiangensis* placed within Clade I (Figures 2 and 3). However, the interspecific relationships within each clade showed some variation depending on the dataset or analytical method used (Figures 2 and 3; Figure S2).

#### 3.3.1 Nuclear Phylogenomic Reconstruction

Phylogenetic trees reconstructed from 45 samples using 1115 nuclear genes and two distinct methods (ASTRAL under the Multi‐Species Coalescent [MSC] model and Maximum Likelihood [ML] in IQ‐TREE) received uniformly high statistical support (Figure 2). Both the coalescent‐based (MSC) and concatenation‐based (ML) nuclear phylogenetic inferences strongly supported the distinct phylogenetic position of *E. yajiangensis* (local posterior probability [LPP] = 1; ultrafast bootstrap support [UFBoot] = 100), despite minor topological differences between them (Figure 2, Robinson‐Foulds distance [RF] = 0.048).

In the MSC tree (Figure 2A), *E. yajiangensis* was positioned as sister to a clade comprising six species: *E. eriocalyx* C.Y.Wu & S.C.Huang, *E. glabra* C.Y.Wu & S.C.Huang, *E. myosurus* Dunn, *E. ochroleuca* Dunn, *E. fruticosa* (D.Don) Rehder, and *E. lamprophylla* C.L.Xiang & E.D.Liu (LPP = 1). In the classic taxonomic system (Wu 1974; Wu and Li 1977), these six species are all classified under *Ser. Fruticosae* C. Y. Wu & S. C. Huang of *Sect. Aphanochilus* Benth. Morphologically, they share several traits with *E. yajiangensis*, such as a shrubby habit, spikes often arranged in lax panicles (equal or nearly secund), and narrow, lanceolate to subulate bracts.

In contrast, the ML tree (Figure 2B) showed a swapped position between *E. yajiangensis* and *E. fruticosa* , rendering *E. yajiangensis* and *E. lamprophylla* as direct sister species.

#### 3.3.2 Plastid Phylogeny

The phylogenetic tree reconstructed from 45 samples using 240 plastid loci also provided strong support for the distinct phylogenetic position of *E. yajiangensis* (UFBoot = 100). Consistent with the findings of Wang et al. (2024), while the three core *Elsholtzia* clades and their interrelationships were fully supported by both nuclear and plastid data, the interspecific relationships within these clades (Clades I–III, Figures 2 and 3) appeared more complex in the plastid phylogeny. In this analysis, *E. yajiangensis* clustered with several populations of *E. fruticosa* , *E. ochroleuca* , *E. glabra* , and *E. eriocalyx* within a single branch. Given the frequent occurrence of hybridization within the genus *Elsholtzia*, the phylogenetic relationships inferred from the plastid genome should be interpreted with caution and serve as a supplementary reference.

### 3.4 Morphological Comparisons and Taxonomy

All species within Clade I exhibit a high degree of morphological similarity. Previous studies have grouped them into the same section, Sect. *Aphanochilus*, based on shared derived characters such as spikes typically arranged in lax panicles (equal or nearly secund), narrow and linear‐subulate to acute bracts, anthers slightly to strongly divergent (but ultimately converging apically), and shiny nutlets. Furthermore, their shared traits of being shrubs, subshrubs, or rarely robust herbs, having lax spikes, large flowers, and slightly exserted, elongated stamens led to their classification within Ser. *Fruticosae*. This series comprises nine species (*Flora of China*; Xiang). Among them, only *Elsholtzia rugulosa* Hemsl. and *E. winitiana* Craib are placed in Clade III, while the remaining seven species, including *E. lamprophylla* described in 2012, collectively form Clade I.

**FIGURE 4:**
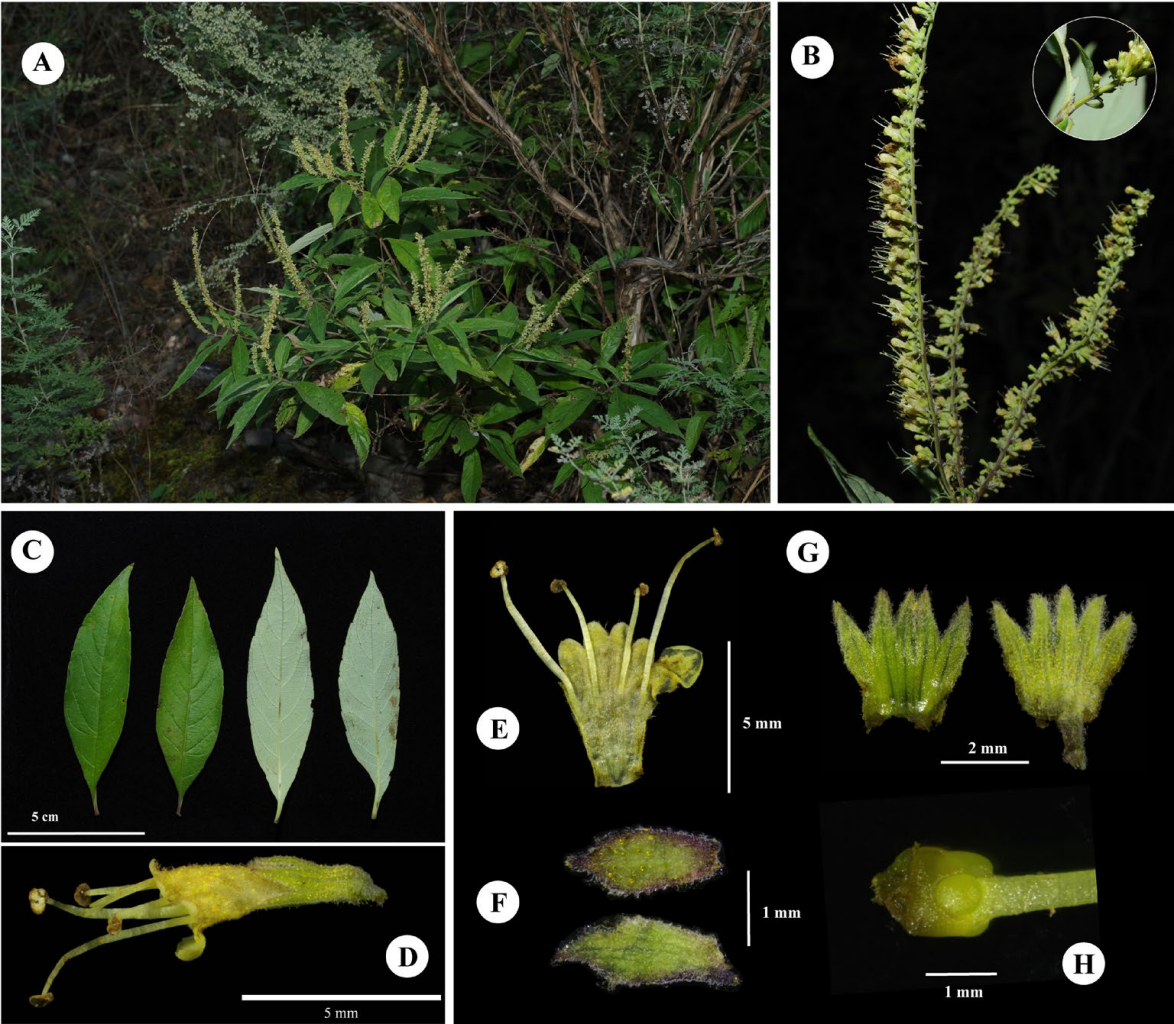
Morphology of *Elsholtzia yajiangensis* sp. nov. (A) Habit. (B) Secund spike showing basal bracts. (C) Leaf. (D) Flower. (E) Dissected flower. (F) Bract. (G) Calyx. (H) Disc and ovary. Photographed by Yan Wang.

**FIGURE 5:**
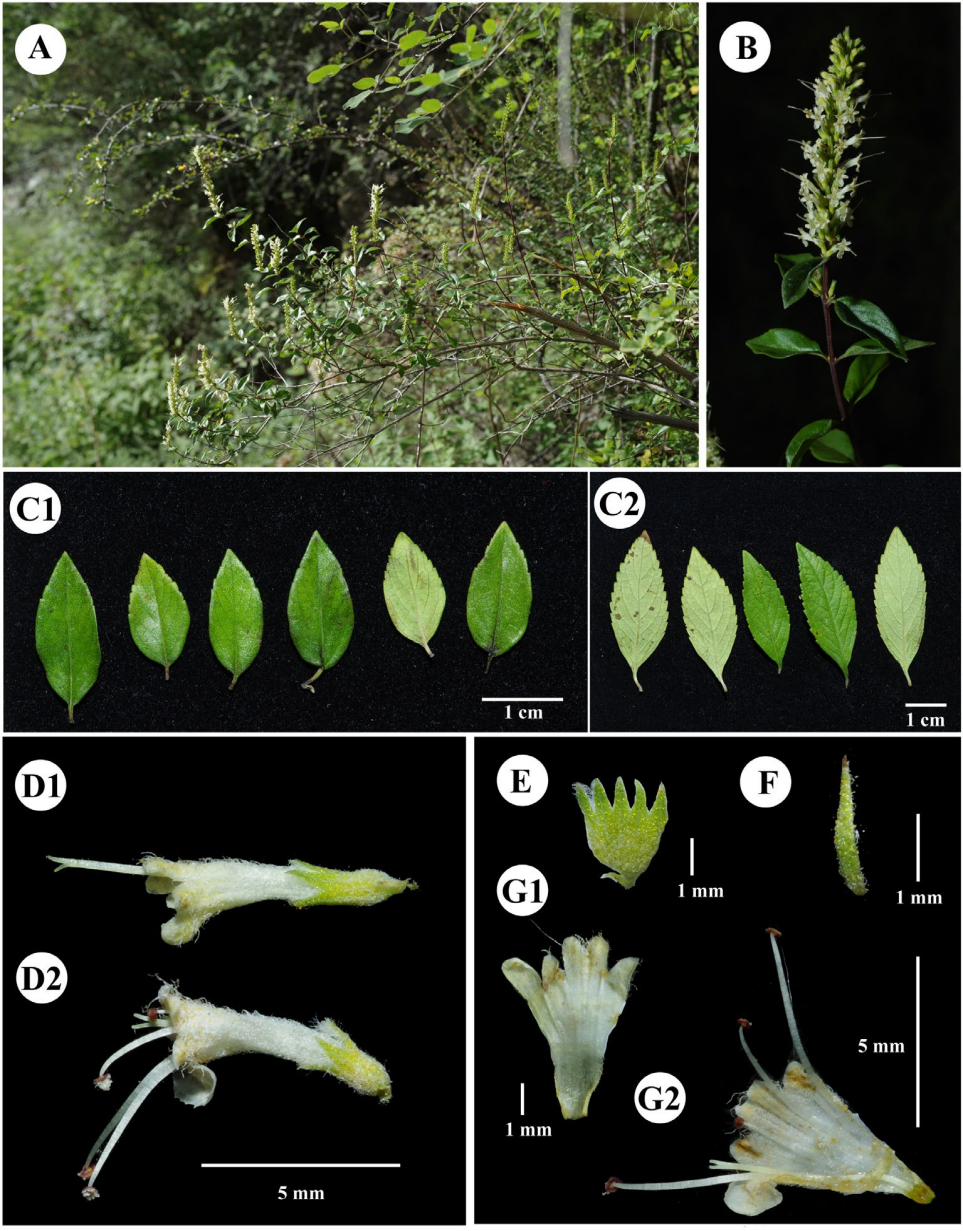
Morphology of *Elsholtzia lamprophylla*. (A) Habit. (B) Cylindric spike. (C) Leaf. (D) Flower. (E) Calyx. (F) Bract. (G) Dissected flower. (1) Flower with aborted stamens. (2) Normal flower. Photographed by Yan Wang.

**FIGURE 6:**
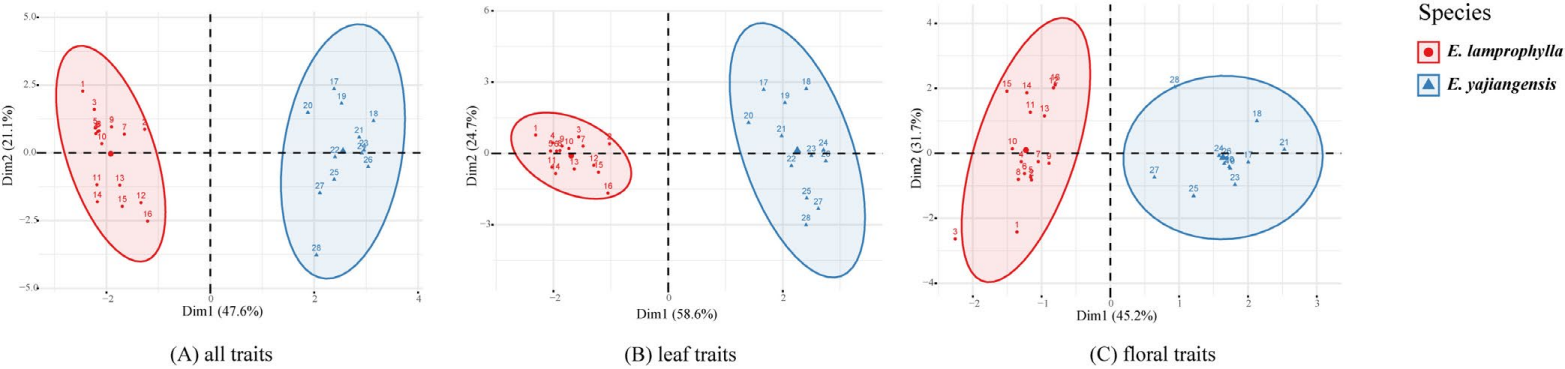
Principal component analysis (PCA) of morphological traits between *Elsholtzia yajiangensis* and *E. lamprophylla*. (A) PCA based on all 12 vegetative and floral traits. (B) PCA based on seven leaf traits. (C) PCA based on five inflorescence/floral traits. Dots represent individual measurements; red = *E. lamprophylla*, blue = *E. yajiangensis*. Ellipses indicate 95% confidence intervals.

Within this group, *E. yajiangensis* and *E. lamprophylla* are morphologically the most similar and were recovered as sister species in the nrDNA ML tree (Figure 2B; UFBoot = 100). However, they can be clearly distinguished by several morphological characters (Table 2; Figures 4 and 5): *E. yajiangensis* has longer spikes (Figures 4A and 5A) that are secund (Figure 4B; vs. cylindrical in Figure 5B); larger leaves (Figures 4C and 5C) with margins entire or remotely obtuse‐serrate (Figure 4C; vs. dentate with small teeth in Figure 5C), and more numerous lateral veins (5–7 pairs in Figure 4C vs. 3–5 pairs in Figure 5C); and broader floral bracts that are broadly lanceolate (Figure 4F vs. lanceolate to linear‐lanceolate in Figure 5F). The PCA results further corroborate the significant morphological divergence between *E. yajiangensis* and *E. lamprophylla* in floral, inflorescence, and leaf traits (Figure 6).

**TABLE 2:** Morphological comparison between *Elsholtzia yajiangensis* sp.nov. and its close relative *Elsholtzia lamprophylla*.

Character/structure	*Elsholtzia yajiangensis*	*Elsholtzia lamprophylla*
Plant height	1.2–1.8 m	0.8–1.0 m
Leaf shape	Elliptic‐lanceolate to oblong	Oval
Leaf size (length × width)	(2.5–)2.8–10.0 × (1.3–)1.5–3.8 cm	0.8–2.0 × 0.3–0.9 cm
Leaf adaxial surface	Green, densely strigose	Sparsely golden glandular and simple‐haired
Leaf abaxial surface	Grayish‐white, with sparse yellow glandular dots	Densely golden‐glandular
Lateral veins (pairs)	5–7	3–5
Leaf margin	Subentire to remotely obtuse‐serrate or crenate	Dentate with small teeth
Inflorescence (spike) length	(4–)6–12 cm	2–9 cm
Lowest bracts	Leaf‐like, subequal to or slightly longer than flowers	Not specified (presumably typical bract‐like)
Floral bracts (bractlets)	Lanceolate to broadly lanceolate, 2–2.5 mm long	Lanceolate to linear‐lanceolate, 2–3 mm long
Corolla color	Yellow	Yellowish to white
Corolla outer surface	With tufted hairs and golden glands	Floccose and golden glandular
Middle lobe of corolla lower lip	Suborbicular, margin recurved and slightly spoon‐shaped, serrate	Circular, margin erose
Stamen length (longer pair)	4.9–6.2 mm	4.5–5.3 mm

It is noteworthy that the character of exserted, elongated stamens is not a reliable diagnostic trait within this clade, as we observed stamen abortion in some populations (e.g., in *E. lamprophylla*, Figure 5D1,G1), which results in inconspicuous stamen exsertion. Despite this observed variability, the combined evidence from morphology, plastid phylogenomics, and nuclear genes consistently supports the recognition of *E. yajiangensis* as a distinct species. Its phylogenetic position within Ser. *Fruticosae*, coupled with the morphological diagnostics summarized above, clarifies its taxonomic boundaries. This study underscores the value of integrating genomic data with detailed morphological observation to resolve species delimitation in complex groups like *Elsholtzia*. Future studies employing population genomics are warranted to explore the cytonuclear discordance observed within Clade I and its potential link to historical hybridization events.

## 4 Conclusion

Integrative evidence from nuclear phylogenomics, plastid phylogeny, and morphology consistently supports the recognition of *E. yajiangensis* as a distinct species within *Elsholtzia* ser. Fruticosae. Despite the relatively limited sampling imposed by the rarity and restricted distribution of the species, both molecular and morphological data clearly distinguish it from its closest relatives, particularly *E. lamprophylla*. The observed cytonuclear discordance within Clade I further highlights the evolutionary complexity of this group and suggests that hybridization may have played an important role in the diversification of Elsholtzia.

The discovery of *E. yajiangensis* also underscores the still underestimated plant diversity of the Hengduan Mountains, a globally recognized biodiversity hotspot and center of endemism for Lamiaceae. The region's complex topography, deep river valleys, heterogeneous habitats, and potential climatic refugia likely promote lineage isolation and local diversification. At the same time, substantial collecting gaps remain in many remote areas, particularly along montane river systems such as the Yalong River valley. Continued integrative studies combining field investigations, morphology, and phylogenomics will likely reveal additional narrowly distributed or previously overlooked taxa in this region.

## 5 Taxonomic Treatment

**FIGURE 7:**
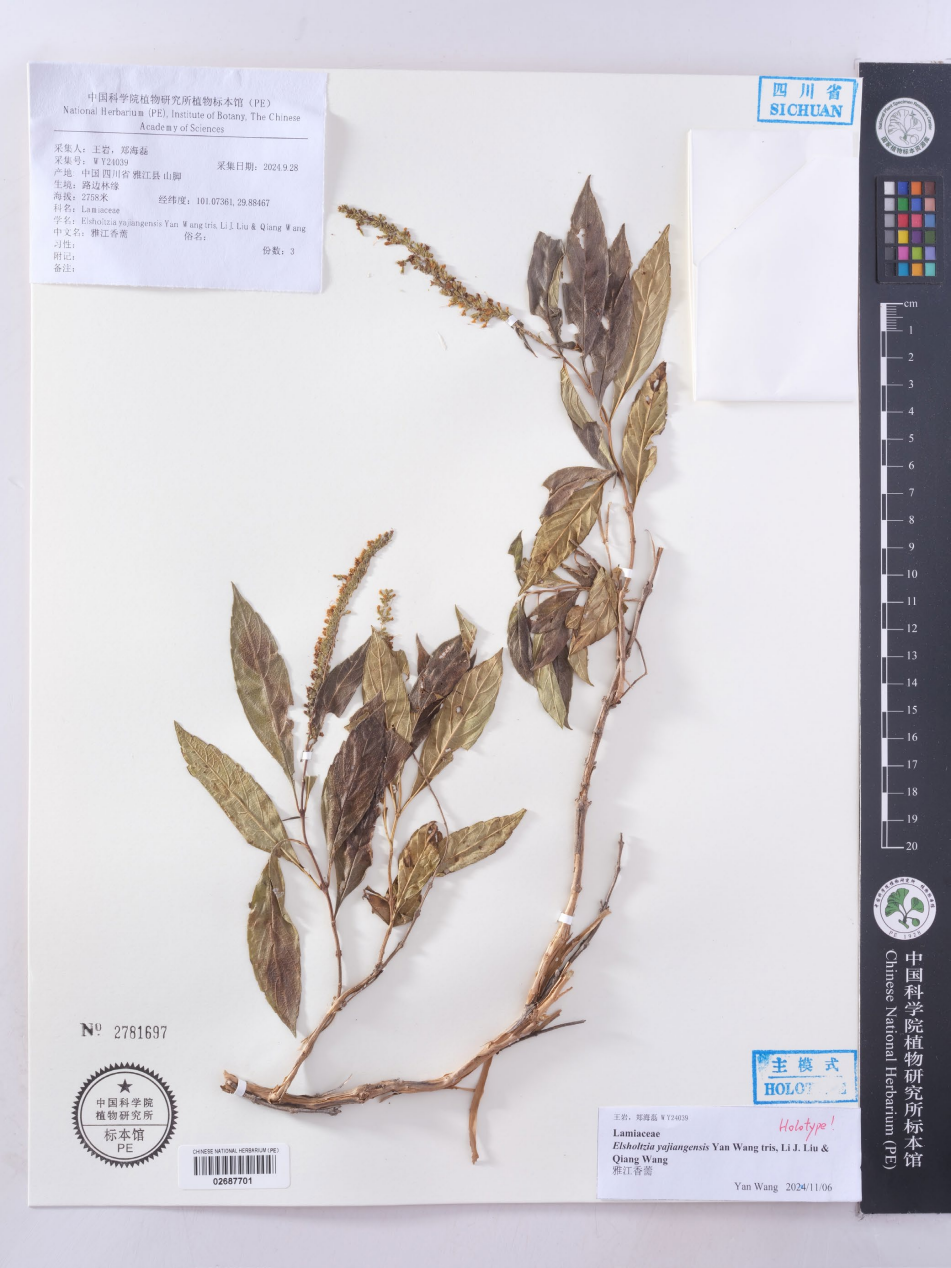
Type of *Elsholtzia yajiangensis* sp. nov. (Holotype PE02687701).

**Elsholtzia yajiangensis Y. Wang, L. J. Liu & Q. Wang, sp. nov**. (Figures 4 and 7)

urn:lsid:ipni.org:names: 77371948‐1

**Diagnosis**. This species belongs to Sect. *Aphanochilus* Benth., Ser. *Fruticosae* C. Y. Wu & S. C. Huang. It is most similar to *E. lamprophylla*, but differs by the following characters: spikes longer (4–12 cm vs. 2–9 cm), secund (vs. cylindrical); leaves larger (2.5–10 cm vs. 0.8–2.5 cm), with margins entire or remotely obtuse‐serrate (vs. dentate with small teeth), indumentum strigose (vs. sparsely glandular and simple‐haired), and lateral veins more numerous (5–7 pairs vs. 3–5 pairs); floral bracts broader, broadly lanceolate (vs. lanceolate to linear‐lanceolate).

**Type**. CHINA. Sichuan Province: Yajiang County, 29°53′4.81″ N, 101°4′24.98″ E, 2578 m, 28 September 2024, *Y. Wang & H. L. Zheng WY24039* (holotype: PE02687701!; isotype: PE02687702‐03!) (Figure 7).

**Description**. Shrubs 1.2–1.8 m tall. Stems 2–8 mm in diameter, glabrous, bark exfoliating longitudinally; branchlets puberulent, green or purplish red when young, turning brown with age. Petiole (0.2–)0.5–1.2 mm, puberulent; leaf blade elliptic‐lanceolate to oblong, (2.5–)2.8–10.0 × (1.3–)1.5–3.8 cm, adaxially green, abaxially grayish‐white, densely strigose and with sparse yellow glandular dots; base narrowly cuneate, margin subentire to remotely obtuse‐serrate or crenate, apex acute; midvein impressed adaxially, prominently raised abaxially, lateral veins 5–7 pairs. Spikes terminal, secund, (4–)6–12 cm long, densely puberulent, with sparse golden glands. Verticillasters 6–10‐flowered, grayish puberulent, densely covered with golden glands. Lowest pair of bracts leaf‐like, subequal to or slightly longer than flowers; floral bracts lanceolate to broadly lanceolate, 2–2.5 × 0.5–0.9 mm, puberulent, with sparse golden glands. Pedicel ca. 0.5–1 mm, inconspicuous. Calyx campanulate, (1.5–)2.5–3 mm, outer surface densely covered with golden glands and villous, inner surface nearly glabrous on the tube, villous on teeth; calyx teeth lanceolate, subequal, 1–1.5 mm. Corolla yellow, ca. 5 mm, outer surface with tufted hairs and golden glands, apex emarginate, inner surface with an oblique, interrupted barbate (beard‐like) ring of hairs near the filament base at mid‐corolla tube; corolla tube ca. 5 mm, base ca. 0.8 mm wide, gradually widening to 1.7 mm at throat; limb 2‐lipped, bilobed, ca. 0.6 mm, lower lip 3‐lobed, middle lobe suborbicular, ca. 2 mm, margin recurved and slightly spoon‐shaped, serrate, lateral lobes semicircular, similar in form to the upper lip lobes. Stamens conspicuously exserted, the anterior pair longer, ca. 4.9–6.2 mm; filaments filiform, glabrous. Style exserted, 6–8 mm, apex 2‐cleft. Disc 4‐lobed, posterior lobe slightly longer, all lobes shorter than nutlets. Ovary 4‐cleft. Nutlets not seen.

**Phenology**. Flowering observed from August to October.

**Distribution and habitat**. This species is currently known only from the type locality in Yajiang County, Sichuan Province, China, where it grows at the foot of mountains, along roadsides, and at forest edges.

**Vernacular name (Chinese name)**. 雅砻江香薷 (yǎ lóng jiāng xiāng rú).

**Etymology**. The specific epithet *yajiangensis* is derived from the type locality Yajiang County in Sichuan Province.

**Note**. According to the current IPNI standard, the author citation of this species would be formatted as “Yan Wang tris, Li J. Liu & Qiang Wang”. However, after careful consideration, we believe that such a citation is unnecessarily long and not convenient for writing, communication, or wider usage. Because Chinese personal names frequently share identical initials when abbreviated into Latin‐letter formats, overlap among standardized author abbreviations is relatively common. Although IPNI generally recommends avoiding duplicate abbreviations, author citations have in practice become increasingly long and complex over time, and this tendency has become more evident in recent years. Furthermore, the International Code of Nomenclature for algae, fungi, and plants (ICN) does not explicitly require author abbreviations to be unique (Madrid Code, Rec. 46). Given that overlaps may still occur during the several‐month review and publication process, we consider a simplified and standardized citation format to be both practical and necessary. As long as the nomenclatural registration (urn:lsid.org) is properly established, the original publication and participating authors remain fully traceable, and the validity and usability of the name are unaffected. Therefore, we adopted the simplified author citation “Y. Wang, L. J. Liu & Q. Wang.”

**Preliminary conservation status**. Data Deficient (DD). The species is currently known from only a single population, which is narrowly endemic to the Yalong River valley in southern Yajiang County at elevations of approximately 2500–2700 m. This results in a small area of occupancy (AOO), and the population lies outside any designated protected areas. Its habitat (road verges and forest edges) is subject to ongoing threats from road maintenance, tourism development, agricultural encroachment, and other anthropogenic activities. These factors are inferred to be driving a continuing decline in the species' area of occupancy, habitat quality, and the number of mature individuals. Further field surveys are urgently needed to assess its full distribution range and population size.

**Additional specimens examined**. CHINA. Sichuan Province: Yajiang County, 2580 m, 25 August 1960, *C. T. Kuan 570335* (KUN 0214814!).

## Author Contributions

**Li‐Jia Liu:** formal analysis (equal), methodology (equal), software (equal), writing – original draft (lead), writing – review and editing (lead). **Yan Wang:** data curation (equal), methodology (equal), project administration (equal), software (equal), supervision (equal), writing – original draft (supporting). **Hai‐Lei Zheng:** data curation (equal), investigation (equal). **Yao Luo:** data curation (equal), investigation (equal). **Hong Jiang:** data curation (equal), investigation (equal). **Qiang Wang:** conceptualization (lead), funding acquisition (lead), project administration (equal).

## Funding

This study was supported by the Science & Technology Fundamental Resources Investigation Program (2022FY202200); the Science and Technology Major Project of Xizang (XZ2025), the Second Tibetan Plateau Scientific Expedition and Research (STEP) program (2024QZKK0200), the Survey of Wildlife Resources in Key Areas of Xizang (ZL202203601 and ZL202303601), and the Youth Innovation Promotion Association, Chinese Academy of Sciences (Y2022032).

## Conflicts of Interest

The authors declare no conflicts of interest.

## Supporting information


**Figure S1:** Circular map of the complete chloroplast genome of *Elsholtzia yajiangensis*. Genes inside and outside the circle are transcribed in opposite directions. Different colors indicate different functional groups. The inner histogram represents GC content variation. The plastome displays a typical quadripartite structure, including the large single‐copy (LSC), small single‐copy (SSC), and two inverted repeat (IR) regions.
**Figure S2:** Comparison between the plastid phylogeny (A) inferred from complete chloroplast genomes using IQ‐TREE 2 and the nuclear phylogeny (B) inferred from nuclear loci using ASTRAL in Elsholtzia. Blue dashed lines connect the same samples between the two topologies. The newly described species, *Elsholtzia yajiangensis*, is highlighted in pink. RF indicates the Robinson–Foulds distance between the two trees.

## Data Availability

All sample data utilized in this study have been uploaded to NCBI. Detailed collection information and accession number are presented in Table 1.
