# Supplementary material for: Elsholtzia yajiangensis (Lamiaceae), a New Species From Sichuan, China
Source: Ecol Evol. 2026 Jun 15;16(6):e73817. doi: 10.1002/ece3.73817 (PMC13266577; doi:10.1002/ece3.73817)

Figure S1. Circular map of the complete chloroplast genome of *Elsholtzia yajiangensis*. Genes inside and outside the circle are transcribed in opposite directions. Different colors indicate different functional groups. The inner histogram represents GC content variation. The plastome displays a typical quadripartite structure, including the large single-copy (LSC), small single-copy (SSC), and two inverted repeat (IR) regions.

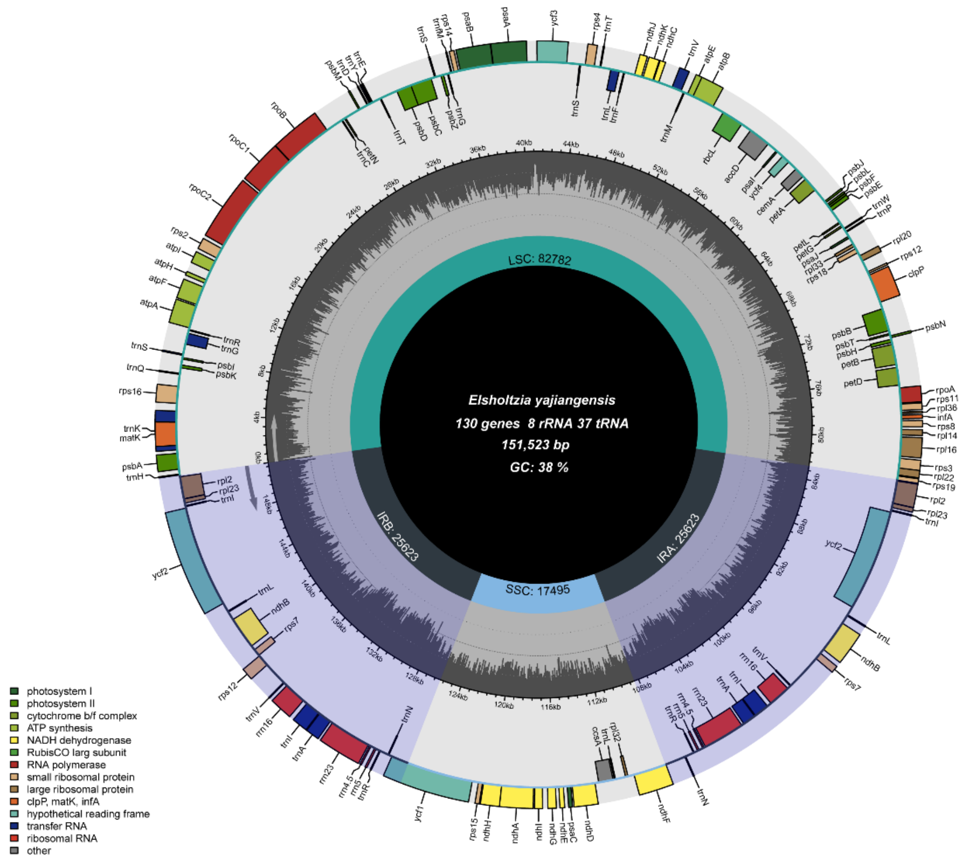

Figure S2. Comparison between the plastid phylogeny (A) inferred from complete chloroplast genomes using IQ-TREE 2 and the nuclear phylogeny (B) inferred from nuclear loci using ASTRAL in *Elsholtzia*. Blue dashed lines connect the same samples between the two topologies. The newly described species, *Elsholtzia yajiangensis*, is highlighted in pink. RF indicates the Robinson–Foulds distance between the two trees.

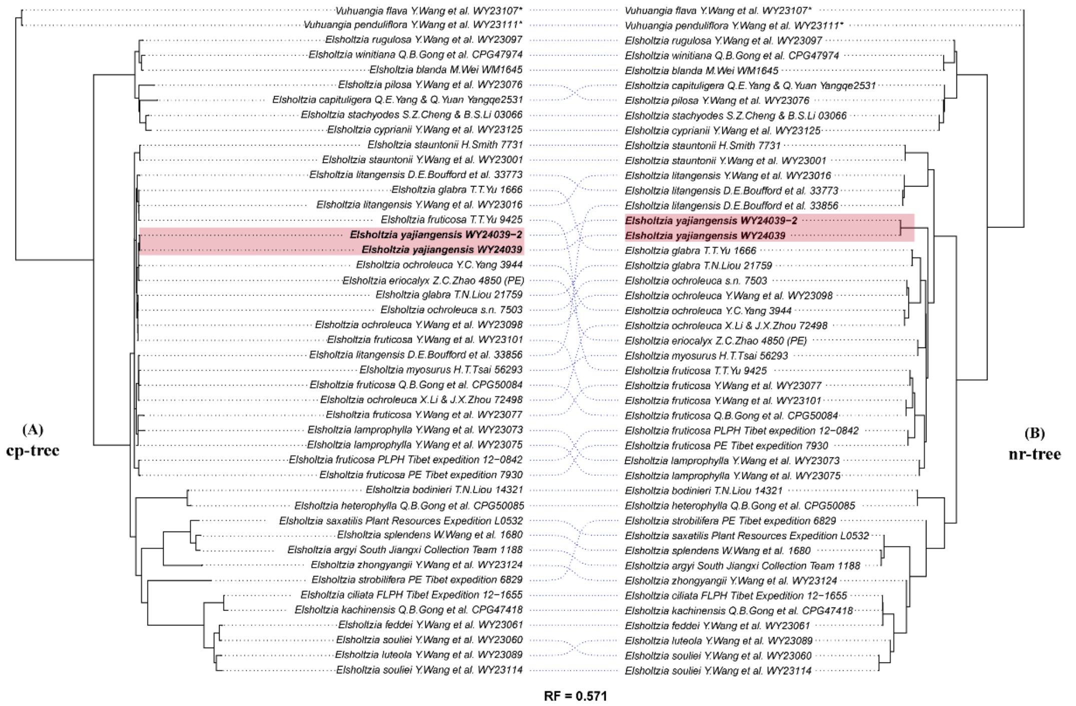

Supplement: Supplementary file 1 — Figure S1: Circular map of the complete chloroplast genome of Elsholtzia yajiangensis. Genes inside and outside the circle are transcribed in opposite directions. Different colors indicate different functional groups. The inner histogram represents GC content variation. The plastome displays a typical quadripartite structure, including the large single‐copy (LSC), small single‐copy (SSC), and two inverted repeat (IR) regions. Figure S2: Comparison between the plastid phylogeny (A) inferred from complete chloroplast genomes using IQ‐TREE 2 and the nuclear phylogeny (B) inferred from nuclear loci using ASTRAL in Elsholtzia. Blue dashed lines connect the same samples between the two topologies. The newly described species, Elsholtzia yajiangensis, is highlighted in pink. RF indicates the Robinson–Foulds distance between the two trees. [file ECE3-16-e73817-s001.pdf]
